# An Ensemble Learning Based Classification Approach for the Prediction of Household Solid Waste Generation

**DOI:** 10.3390/s22093506

**Published:** 2022-05-05

**Authors:** Abdallah Namoun, Burhan Rashid Hussein, Ali Tufail, Ahmed Alrehaili, Toqeer Ali Syed, Oussama BenRhouma

**Affiliations:** 1 Faculty of Computer and Information Systems, Islamic University of Madinah, Madinah 42351, Saudi Arabia; alrehailiium@iu.edu.sa (A.A.); toqeer@iu.edu.sa (T.A.S.); oussama.benrhouma@iu.edu.sa (O.B.); 2 School of Digital Science, Universiti Brunei Darussalam, Tungku Link, Gadong BE1410, Brunei; 18h8338@ubd.edu.bn (B.R.H.); ali.tufail@ubd.edu.bn (A.T.); 3 Department of Informatics, University of Sussex, Brighton BN1 9RH, UK

**Keywords:** solid waste generation, smart waste management, forecasting, time-series, machine learning, ensemble learning, meta learner, smart cities, IoT

## Abstract

With the increase in urbanization and smart cities initiatives, the management of waste generation has become a fundamental task. Recent studies have started applying machine learning techniques to prognosticate solid waste generation to assist authorities in the efficient planning of waste management processes, including collection, sorting, disposal, and recycling. However, identifying the best machine learning model to predict solid waste generation is a challenging endeavor, especially in view of the limited datasets and lack of important predictive features. In this research, we developed an ensemble learning technique that combines the advantages of (1) a hyperparameter optimization and (2) a meta regressor model to accurately predict the weekly waste generation of households within urban cities. The hyperparameter optimization of the models is achieved using the Optuna algorithm, while the outputs of the optimized single machine learning models are used to train the meta linear regressor. The ensemble model consists of an optimized mixture of machine learning models with different learning strategies. The proposed ensemble method achieved an R2 score of 0.8 and a mean percentage error of 0.26, outperforming the existing state-of-the-art approaches, including SARIMA, NARX, LightGBM, KNN, SVR, ETS, RF, XGBoosting, and ANN, in predicting future waste generation. Not only did our model outperform the optimized single machine learning models, but it also surpassed the average ensemble results of the machine learning models. Our findings suggest that using the proposed ensemble learning technique, even in the case of a feature-limited dataset, can significantly boost the model performance in predicting future household waste generation compared to individual learners. Moreover, the practical implications for the research community and respective city authorities are discussed.

## 1. Introduction

The world’s population is increasing at a staggering rate, and consequent challenges are already evident in different sectors. Currently, approximately 56% of the world’s population reside in urban regions, and this percentage is anticipated to rise to 68% by 2050 [1,2]. Many challenges can arise with such an extraordinary influx and settlement of people in urban areas. One of these challenges relates to the management of solid waste generation in urban cities. On average, the world generates roughly two billion tons of waste every year, and one person generates, on average, 0.74 kg of solid waste per day [3]. This can impose a huge burden on the waste management authorities, especially in light of the limited resources and infrastructure and associated costs [4]. To reduce the adverse waste generation implications, several implementation instances were developed, fusing a myriad of modern technologies, ranging from IoT-based architectures [5] and sensing devices [6] to computational intelligence [7]. Despite these efforts [8], large cities constantly need new techniques and strategies to optimize their waste management activities.

The process of waste management comprises multiple sequential activities, including waste disposal in the containers by the citizens, waste collection by the truck fleet, and finally, waste transportation to the designated waste landfills or recycling centers [5]. These activities implicate a substantial cost, particularly when performed in a manual way. Another observation is that the impact of this cost is more profound in developing countries than in developed countries. Some factors that aggravate the situation include the lack of clear waste disposal policies and the inability to deploy modern scientific systems and methodologies [9]. Several researchers advocate the integration of information and communication technology solutions to carry out waste management activities more economically [5,10,11,12]. The use of smart waste management techniques is not just about deploying waste collection but also extends to optimizing municipality resources, implementing such processes efficiently, and promoting sustainable and environment-friendly attitudes and practices through the application of modern machine learning classifiers. 

Recently, machine learning (ML) has been incorporated into waste management systems. One example of such an application can be found in [13], where the authors combined artificial intelligence and IoT to enhance the performance and dynamically control waste management processes in the city of Copenhagen in Denmark. Moreover, in order to efficiently collect and dispose of the waste, the study suggested using a combination of multiple techniques, including the shortest path spanning tree, graph theory, k-means, geographic information system, and genetic algorithm. The authors claimed that their proposed solution decreased the bin overflow by four-fold. In a similar study [14], the authors proposed an IoT based waste monitoring architecture that utilizes Raspberry Pi and ultrasonic sensors to help gather waste related data. The sensor data is used to prepare a waste collection schedule to collect the trash in a timely and resourceful manner. The authors claimed that a whopping 46% reduction in fuel cost was achieved by deploying their proposed architecture. Moreover, waste collection trips were reduced by 18%. However, the findings are not reliable since the data is based on merely ten days of testing. Also, no ML approaches have been devised to predict the waste level of the bins and containers accurately. In yet another study [15], the authors employed advanced techniques, e.g., neural networks and decision trees, to estimate the solid waste generated while taking advantage of the demographic and socioeconomic characteristics of the citizens of Ontario (Canada). The accuracy of predicting waste generation by utilizing the neural network technique was 72%, which suggests that the prediction accuracy needs to be improved.

### 1.1. Motivations

The concept of smart waste management (SWM) [11,16] is a fairly new and topical research challenge that has received considerable attention from municipalities and the research community because of its economic and environmental importance [17]. Several research studies explored smart waste management from various angles, e.g., IoT infrastructure and predictive analytics [18,19]. Despite the increasing number of projects investigating intelligent computational models to overcome the challenges of waste management, more research works are advocated to exploit the benefits of IoT infrastructure and solutions to enhance ML based waste management models in municipalities and urban regions [18]. In a recent survey [18], only six predictive models out of 32 were found to create intelligent solutions for handling solid waste management. In addition, another study emphasized that waste data collection alone is insufficient to tackle the waste management issues [14]. It is highly advisable to conduct predictive analytics to develop effective SWM solutions.

Several recent works developed predictive analytics and AI-based models to eliminate urban waste management issues and improve the existing SWM solutions. For instance, the authors in [20] created a decision tree model that effectively calculates the number of waste bins awaiting waste truck collection. The results demonstrated a decrease in the distance of collection routes proposed by the model. The authors in [21] developed a recurrent neural network (RNN) classifier to forecast the waste bins’ fill levels. The RNN model was tested in a Spanish city and produced better fill level predictions than existing ML approaches. Moreover, the authors in [22] developed a deep learning classifier (DL) to segregate and sort solid waste in a smart way. The TensorFlow DL was first fed LoRa communicated sensor data. Next, the model detected and classified the waste objects into different types (glass, metal, plastic, etc.). The classification results showed different sorting accuracy of waste (82ߝ96%) based on solid waste materials. In our view, however, the proposed system is over-engineered and expensive to apply in real settings (i.e., municipalities). The system focuses mainly on waste identification and segregation rather than the optimization of waste authorities’ resources. Furthermore, there is a lack of predictive models tailored to handle time-series waste generation problems [23]. In this research, we explore how various ML models (i.e., single and ensemble) can forecast the waste generation of citizens and municipalities. 

We conducted a comprehensive review of the research works, published post 2015, in the sector of smart waste management to identify the overarching challenges in SWM. The full results of the review are presented in Section 2 (Related Works). We highlight the practical and research issues pertaining to intelligent waste management solutions and their applications as follows:

- Poor use of smart waste management solutions in municipalities: in numerous urban cities around the world, the acceptance and use rate of new technologies (e.g., ML) that assist in the management of solid waste (e.g., waste collection, recycling) is still low.
- Inefficient and expensive IoT devices: transforming any city into a smart city, sadly, comes with a high financial and environmental cost. The practical challenge for municipalities is to reduce the reliance and installment of IoT sensors and devices while augmenting the waste management sector. ML techniques might offer the necessary escape route.
- Unoptimized use of waste management resources: legacy computer systems burden the capacity and resources (e.g., collection trucks, human capital) of waste management authorities. Examples of disadvantages include, but are not limited to, the inefficient waste bin collection routes and failure to meet the expectations and needs of the citizens.
- Lack of predictive analytics for waste management (SWM): municipalities are required to monitor in real-time and predict the waste of their cities (e.g., waste containers fill levels) during the season and thereby deploy the necessary infrastructure to accommodate the changing demands of the city households.
- Upgradation of waste management solutions with smart IoT architectures: waste management authorities that are using legacy platforms are in dire need to modernize their infrastructure to incorporate smart waste sensors to supply predictive models with the necessary waste data.
- Need for sustainable waste management attitudes and behaviors: there is an urging need to innovate intelligent waste management solutions that promote sustainable waste disposal practices and engage households in the reduction and recycling of their solid waste.

Problems are better elaborated when contextualized. An ordinary person is anticipated to generate about 0.74 kg of solid waste per day. Nonetheless, this figure can vary between 0.11 kg and a whopping 4.45 kg per person per day, depending on various socioeconomic factors. Moreover, the amount of solid waste is predicted to exceed 3.4 billion tons worldwide by the year 2050. Several factors are believed to contribute to the waste generation problem, including the economic status of the country, household income, demographics, season of the year, etc. In fact, many countries, developed and developing, still struggle with managing and disposing of their solid waste effectively. The costs and resources required to perform waste management activities remain expensive (e.g., averaging USD 100 per ton), with a high percentage expended on waste transportation (approximately USD 20 to 50 per ton). Such statistics urge practitioners and researchers to accelerate their SWM efforts and innovations. Creating data-driven models that are capable of predicting household waste generation accurately in urban cities would help the authorities plan their waste management activities more efficiently and cut their relative spending. The expected benefits of predictive analytics in waste management are manifold, e.g., reduced reliance on IoT devices, optimal use of local resources, and increased citizens’ satisfaction, among others.

### 1.2. Main Contributions

We succinctly summarize the notable contributions of our research study as follows:

- We proposed designing and training an ensemble model that can predict solid waste produced by the citizens/households of an actual city.
- We employed the Optuna algorithm, which uses an efficient sampling and pruning strategy to optimize the hyperparameter configurations to maximize the predictions of waste generation for all single ML models.
- To the best of our knowledge, we are the first study to introduce a meta regressor as an ensemble technique that learns from the predictions of the optimized machine learning models for forecasting household waste generation.
- We validated our ensemble meta learner model using a real dataset with limited features to make significant time-series predictions.
- We compared the proposed method and benchmarked it against existing state-of-the-art methods for predicting household waste generation.

### 1.3. Article Structure

The remainder of this article is divided into five sections. Section 2 reports on the recent works in the domain of smart waste management with a focus on the prediction of waste generation using machine learning. Section 3 describes our methodology and the time series dataset that was used in our experiments. Section 4 details and discusses the main results of the benchmark experiment. Section 5 discusses the theoretical and practical implications of our findings. Finally, Section 6 summarizes the key findings and suggests future research directions.

## 2. Related Works

Several published studies explored machine learning approaches for forecasting waste generation. These studies have been conducted on data collected from different cities with multiple features, as described below.

For instance, Ferrer and Alba [24] proposed an smart system (aka BIN-CT) for predicting future filling levels of waste containers. The study collected several features, such as container location, time, container capacity, specific day, and whether the container was emptied the day before, from Andalusia city in Spain for twelve months. Different machine learning models, e.g., support vector regression (SVR), linear regression (LR), and Gaussian processes (GP) were evaluated. The authors suggested using GP as it performed better than other algorithms. Similarly, Camero et al. [25] proposed a deep neuroevolutionary method to create a recurrent neural network for predicting waste filling levels from waste containers in an automatic manner. The search space included different parameters, for instances the number of hidden layers and neurons, which were optimized using an evolutionary algorithm. The authors applied the optimized model to a similar dataset used in Andalusia city, Spain [24], to predict waste filling levels of 217 containers. The authors achieved a mean absolute error (MAE) of 0.028 for the summed-up prediction. However, another study [26] presented a neural network model that can be utilized to predict plastic waste generation for the European Union region in 2030. The main focus of the study is to avert any environmental hazard by controlling plastic waste generation. Authors in [27] deployed a multi-layer perceptron artificial neural network model (aka MLP-ANN) and support vector regression (SVR) model to forecast solid waste of the municipalities of the Kingdom of Bahrain. By utilizing the waste generation data for 12 years, the authors showed that the SVR is more robust in predicting municipal solid waste. The factors that were considered in the prediction incorporated the economic level, population, annual tourist arrivals, and CO_2_ emissions.

Other studies have further investigated the impact of socioeconomic and other demographic factors when predicting waste generation levels. For example, Kontokosta et al. [28] proposed a machine-learning-based approach to estimate daily and weekly waste generation at a building scale. The study used daily historical records collated from 609 sub-sections of New York City (USA) over ten years and other demographic and weather data of the population. The study implemented a gradient boosting regression tree (GBRT) and reported a good R-squared value (i.e., 0.87) when forecasting the weekly waste generation in New York City. The author also reported the weather variables and building type and density as the most influential features for weekly waste generation predictions. Similarly, Yang et al. [29] proposed a novel predictive approach to unravel the effect of socioeconomic characteristics on solid waste generation. The study collected annual statistical waste generation data and nine socioeconomic factors of all provinces in mainland China between 2008 to 2019. However, weather data were excluded from the analysis. Different machine learning models were contrasted, including k-nearest neighbors (KNN), support vector regression (SVR), random forest (RF), XGBoosting, and a deep neural network (DNN). DNN outperformed other classifiers, with an R-square value of 0.97. The authors reported that indicators such as urban population and regional GDP had the most influence in predicting MSW generation for different provinces in China.

On the other hand, Kulisz and Kujawska created an ANN model to predict the future waste generation of Polish cities [30]. The study included five features, namely social, economic, and demographic factors, to estimate the amount of annual waste generated from 2010 to 2019. The study concluded that the city’s population was the most influencing factor in waste generation, with the proposed ANN achieving an R-value of 0.989. In another study, Flores et al. [31] tried to determine the relationship between socioeconomic and demographic variables. They claim that this relationship helps to estimate the type and amount of annual solid waste production per country. To predict waste generation, the authors deployed various machine-learning-based approaches, such as SVR, gradient boosting, RF, and clustering models (k-means). However, their work focused on Organization for Economic Co-operation and Development countries (OECD).

Similarly, Elshaboury et al. [32] used the same features. Further, they proposed optimizing the ANN with an evolutionary particle swarm optimization (PSO) algorithm to forecast waste generation in the Poland cities, with a reported RMSE of 11,342.74. Kannangara et al. [15] also used similar demographic and socioeconomic variables to predict MSW generation and diversion from Ontario, Canada. Using data from 220 municipalities from 2001 to 2014, the authors applied the decision tree (DT) and ANN to calculate the annual waste generation. The ANN model surpassed the DT, with a 0.72 R-squared value on the test data. To predict future waste generation, Rathod et al. [33] compared different machine learning algorithms, such as LR, DT, RF, and XGBoost. The study collected data from 200 areas in the Akola municipality (India) using various demographic and socioeconomic features. The authors demonstrated that RF was the best-predictive model, with an adjusted R2 value of 0.6758.

Furthermore, Meza et al. [34] employed a similar approach by evaluating various machine learning algorithms, including SVM, RNN, and DT, to predict urban waste generation. Historical monthly generated solid waste data between 2012 and 2016 were fed into the model, with various demographics and socioeconomic factors collected from Bogotá (Colombia). The results showed that SVM was the best alternative model for future waste generation prediction.

Other studies inspected the forecasting of waste generation as a time series problem. For example, Abbasi and Hanandeh [35] evaluated different machine learning models, including a neural network (ANN), support vector machine (SVM), k-nearest neighbors (K.N.N.), and adaptive neuro-fuzzy inference model (aka ANFIS) to forecast the monthly trends of municipal waste generation in the city of Logan (Australia). Historical time series data covering eighteen years were used to train these three classifiers for making future month predictions. The results suggested that the ANFIS model outperformed both KNN and SVM, with an MAE of 52.16. On the other hand, Sunayana et al. [36] proposed using a non-linear auto regressor (AR) neural model to predict the monthly waste generation from Nagpur (India). Historical monthly waste generation data were used from the year 2015 to 2017. The study used lag estimation in developing a robust model for future waste prediction, with the best-optimized model resulting in a mean absolute percentage error (MAPE) of 2.37. Ali and Ahmad [37] also employed a non-linear neural model to forecast MSW generation. The authors collected monthly waste-generated data from Kolkata (India) from 2010 to 2017. The authors optimized the network using a grid search, where they varied different neurons in the input layer. A 0.8643 R-value was reported as the highest performing network when using only 19 hidden neurons.

Internet of Things (IoT)-based infrastructure has been widely utilized for providing various intelligent solutions in many domains. Similarly, recent research works advocate the deployment of IoT to ameliorate waste management processes. For example, the authors in Baldo et al. [38] presented a long range wide area network (LoRaWAN)-based architecture, where they presented three layers of implementation, starting from the smart bins level to the video surveillance level. Similarly, the authors in [39] discussed an IoT-enabled waste management solution that monitors the fill levels of the bins in public and residential premises. With the help of the graphical user interface and location information, authorities could dispatch waste collection trucks to collect the solid waste. Most of these studies do not consider the analysis of waste generation using ML approaches, e.g., neural networks, deep learning, etc.

Internet of Things (IoT) is the next-generation technology that is revolutionizing almost every sphere of human life, ranging from industrial automation, bioinformatics, smart homes, smart cities, vehicle automation, virtual assistants, virtual/augmented reality, etc. [40,41]. IoT technologies are based on multiple physical devices that are connected to the Internet and help make seamless decisions, mostly without human intervention, by sensing, collecting, and sharing valuable data [5].

Quite recently, the combination of IoT and machine learning techniques has been widely explored, with the aim of providing automated services in waste management, including but not limited to bin level prediction, waste collection, processing, and dumping [42,43,44]. With the help of IoT sensing devices and appropriate AI-based approaches, bins fill levels can be predicted and monitored by waste collection authorities to optimize their daily processes, such as dispatching collection trucks [45]. Additionally, IoT-AI-based waste collection can assist in the calculation of the most efficient routes to collect the waste, thus reducing the overall cost and preserving the environment by cutting associated CO_2_ emissions [43]. The aforementioned efforts demonstrate the importance of IoT in future research and applications related to intelligent waste management.

From the surveyed literature, we can clearly see that no single machine learning algorithm has performed consistently across the solid waste data. Furthermore, most studies relied extensively on the additional features, e.g., socioeconomic factors and population demographics, to predict future waste generation rates. Although it is practical to use these features in the prediction, such sensitive data are usually unavailable or may be hard to obtain. Studies performed on different cities show that access to time series waste data is still limited and, therefore, it is hard to benchmark the proposed approach on other datasets. Finally, deep learning approaches have been commonly used, with promising performances among the algorithms. This study proposes an ensemble learner to predict weekly household waste generation based on historical waste data collected from actual households. Details of the proposed approach are explained in the subsequent sections.

Table 1 highlights some of the important studies, along with a brief summary of the strengths and weaknesses of each study. This will help the reader to obtain an overview of waste generation and prediction, particularly where approaches, such as ANN and ML, have been utilized. The weaknesses highlight the overall gaps in this field. However, our research focuses on predicting household waste generation using time series data.

## 3. Materials and Methods

**Figure 1:**
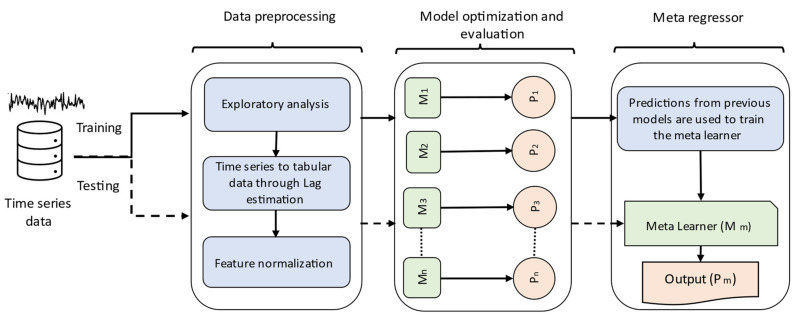
The proposed method for the prediction of solid waste generation (in model optimization phase: M = model, P = prediction).

Figure 1 depicts the proposed methodology for developing a predictive ensemble model to forecast future waste generation. Our method consists of three phases, where each phase represents a separate component, namely the data preprocessing, model optimization, and meta regressor component. Details of each component are described in the subsequent sub-sections. While single prediction models have been widely used in the literature, ensemble learning has demonstrated its ability to enhance the performance of individual ML models and is more robust to noise [46,47]. In the proposed method, several machine learning algorithms are trained and optimized before making their predictions. The output of these single ML models is then used to train a linear model (aka Meta learner) for the final prediction.

As shown in the Section 4, this approach enables the framework to learn residual errors of individual models and thereby improve the final model prediction. Algorithm 1 describes the pseudocode of our proposed ensemble model.

**Algorithm 1.** Pseudocode for training the meta classifierInput: Training Dataset $D,=\left\{\;\left(x_{i},\;y_{i}\right)\;\in \chi \;\subseteq {\mathrm{IR}}^{d}\times Y\;:1\;\leq \;i\;\leq \;N\;\right\}$Output: An ensemble classifier *C* for weekly waste generation
1:  Step 1: Train the first-level classifiers2:  **For** *m* ←1 to *M* **do**3:          Train a base classifier *c_t_* using the solid waste dataset *D*4:  **End For**5:  Step 2: Create a new dataset from the output of first-level classifiers with *D* labels6:  **For** *i* ←1 to *N* **do**7:         Create a new waste dataset containing $\left(x^{≀}{}_{i},\;y_{i}\right)$ where $x^{≀}{}_{i}=\{\;(c_{1}\left(x_{i}\right),(c_{2}\left(x_{i}\right),\;\ldots \;(c_{t}\left(x_{i}\right)\}$8:  **End For**9:  Step 3: Train a meta classifier10:Train a new classifier *c*′ using the newly developed solid waste dataset11:
**return** $C(x)=c^{\prime }\left(c_{1}\left(x\right),\;c_{2}\left(x\right),\;\ldots \;c_{T}\left(x\right)\right)$




### 3.1. Solid Waste Dataset

The solid waste dataset used to train and assess the ML models in this research was obtained from the Southeast region of the United Kingdom (UK), representing an average weekly household waste generation from 2011 to 2021. The dataset, model implementation, and prediction results were deposited into a public repository on GitHub [48]. The dataset contains the weekly waste generation data per region in the UK. The dataset provides values for the ‘Year’, ‘Region’, and ‘Collected Household Waste’. A total of nine regions were included in the dataset. We selected the data of the Southeast region for the purpose of preprocessing, training, validating, and testing. The dataset can be considered a small dataset. However, it provides a good insight into the waste generation patterns of UK households. A small dataset can sustain issues in supervised learning scenarios. This kind of dataset is termed a low-quality data problem [49]. Due to the lack of open and comprehensive solid waste generation data, we utilized the abovementioned data set. Moreover, after carefully preprocessing, training, validating, and testing, we found it useful for analysis purposes.

### 3.2. Data Preprocessing

*Even* though the above dataset is represented as a time series domain, we reformulated the dataset as a supervised learning problem by using the lag estimation as an input feature to train supervised regression models. Lag estimation or selection is a classical technique that helps to transform the forecasting problems into a supervised learning problem [50,51]. The lag value is known to help improve the prediction accuracy. The selection of this value is critical since a small lag value can affect the prediction by providing insufficient information, whereas a large lag value can increase the complexity and, at the same time, decrease the performance and accuracy of the prediction [52]. For this reason, the number of lags was experimentally found based on the validation set’s performance used in building the models. After selecting appropriate lag values to use as input features, the dataset was divided into 60% training, 20% validation, and the remaining 20% for testing. The data were then normalized to a uniform range of 0–1 using Equation (1) to suppress the large values for improving model training and performance.

(1) $${𝒰}_{i}=\frac{\left(R_{i}-Min_{i}\right)}{\left(Max_{i}-Min_{i}\right)}\;$$

where ${𝒰}_{i}$ is a normalized input, $R_{i}$ is the actual input value, $Min_{i}$ is the minimum value of the input data, and $Max_{i}$ represents the maximum value of the input dataset.

### 3.3. Machine Learning Algorithms

An exhaustive list of various machine learning algorithms was devised and used to predict solid waste generation [53]. Notably, the performance of these algorithms depends on the characteristics of the dataset being used. Hence, no ideal algorithm performs well across all problems [54]. For instance, Sami et al. [55] employed a SVR, RF, and decision tree as traditional machine learning algorithms, while CNN was utilized as a deep learning model in predicting solid waste management. In the employed models, CNN outperformed the other models, with an accuracy of 90%. However, the accuracy of the other machine learning approaches, as yielded by SVM, RF, and DT, was considerably less, i.e., 85%, 55%, and 65%, respectively.

In another work, Xia et al. [53] reviewed around 200 research publications spanning from 2000 to 2020 and compared various machine learning approaches employed to address the fast-growing waste management issue. The selected ML and deep learning techniques regarding the prediction of waste collection include the use of LSTM, ANN, SVM, DT, etc. Based on the presented results, the prediction of the RSME of the LSTM was 0.5 [56], whereas SVM presented an RSME of 9.53 in [57], 0.98 in [35], and 0.12 in [58]. The ANN yielded an RSME of 0.87 in [28], 0.75 in [57], and 0.50 in [15]. The accuracy results pertaining to the DT prediction was 0.77 [28]. These findings exhibit considerable variability in the scores of performance metrics across various studies.

Therefore, in this study, we experimented with a set of machine learning algorithms that have been commonly used for predicting waste generation. These algorithms include SVR, RF, artificial neural network (ANN), and KNN [55]. Furthermore, our study included other machine learning algorithms that are less popular in waste management but have shown a promising performance when applied to other domains. These algorithms include extra gradient boosting (XGBoosting), light gradient boosting machine (LightGBM), and extra trees (ETS) [59].

Table 2 presents a conceptual comparison of the single prediction algorithms that were inspected in our study. It highlights the strengths and weaknesses of each model.

### 3.4. Model Hyperparameter Optimization

Hyperparameter optimization is a major challenge in the ML community [60]. This step involves finding an appropriate hyperparameter configuration that ameliorates the model’s performance for a given dataset. Model hyperparameters are usually set before the learning process and are tuned based on the performance of the selected hyperparameter on a validation set performance as an objective. Most existing hyperparameter optimization algorithms, such as the grid search, are computationally expensive since they require searching for all possible defined hyperparameter configurations to identify and select the best model [61]. Other algorithms, such as the random search, try to overcome the limitations of the grid search by optimizing the model in a randomly selected hyperparameter configuration, but may result in a suboptimal hyperparameter configuration due to its stochastic nature [61].

Bayesian optimization algorithms provide an alternative solution by developing a surrogate model that helps to select the next most promising hyperparameters from the search space based on the previous evaluations [60]. A recent state-of-the-art hyperparameter optimization algorithm, “Optuna”, has been used for this purpose [62]. Unlike existing hyperparameter optimization (for example, hyperOpt, scikit-optimizer, Autokeras), Optuna allows for a dynamic construction of the hyperparameter search space with an efficient sampling and pruning strategy. This strategy is rewarding for achieving a high performance under limited resources [62]. Optuna considers a minimization/maximization of an objective function as the input with a given hyperparameter search and returns the validation score as the output. A detailed discussion of the Optuna algorithm can be found in the original work published by [62]. Apart from the meta regressor model, all other machine learning models proposed in this study were optimized using Optuna. Table 3 below summarizes the hyperparameter configurations that were used to maximize the predictions of all single ML models using Optuna.

### 3.5. Meta Regressor

The meta regressor [63,64] is a simple linear regression model trained using the predicted output of individual optimized models. The training samples are first passed to individual optimized models to obtain the model predictions to train, in the subsequent phase, the meta regressor model. The outputs of these models are then transformed by horizontal stacking individual model predictions to formulate tabular data. These data are then used as input features to the regression model to perform the training process. During testing, a similar procedure is applied to a hold-out validation set. It is first passed to individual models to obtain their predictions, followed by the data transformation, and is finally passed to the meta regressor model to obtain the final prediction.

### 3.6. Model Evaluation

When evaluating machine learning models, it is essential to select an appropriate metric to be able to identify and select the best-performing model. Many standard evaluation metrics are proposed and commonly used to judge the quality of forecasting models [65,66]. Several metrics are used with various properties in this study to compare the ML models fairly. Below is a summary of the metrics considered for this study.

#### 3.6.1. Mean Absolute Error (MAE)

The mean absolute error metric [67] considers the sum of the absolute difference between the actual value and the predicted value. The MAE is less biased to higher values and, hence, is considered more robust when dealing with outliers. The MAE is also considered a linear score since it gives an equal weight to individual errors. The unit score matches that of the target values and, hence, becomes easy to interpret. It typically ranges from 0 to ∞, where a small value close to 0 is considered the best score. Equation (2) shows how the MAE is calculated.

(2) $$\mathrm{MAE}=\frac{1}{n}\;\sum_{i=1}^{n}|y_{i}-{\hat{y}}_{i}|\;\;\;\;\;\;\;\;\;$$

#### 3.6.2. Mean Squared Error (MSE)

The mean squared error (MSE) metric [68] represents an average of the squared differences between the actual and predicted values. The MSE can be used as a metric score and loss function when optimizing models by minimizing the MSE in the least-squares regression problem. When used as an evaluation metric, squaring off the error magnifies the error and penalizes the model by inflating the average score. On the other hand, the squaring of errors tends to penalize the model more on significant errors when used as a loss function. The metric has a squared unit and ranges from 0 to ∞, where a value close to zero is the best score. Equation (3) shows how to calculate the MSE of a given dataset. The root mean squared error (RMSE) extends the MSE by taking the squared root of the MSE. As such, it has the same unit as the actual values and ranges from 0 to ∞, where the value close to zero represents the best score. Equation (4) shows how to calculate the RMSE of a dataset.

(3) $$\mathrm{MSE}=\frac{1}{n}\;\sum_{i=1}^{n}{\left(y_{i}-{\hat{y}}_{i}\right)}^{2}\;\;$$

(4) $$\mathrm{RMSE}=\;\sqrt{\frac{\sum_{i=1}^{n}{\left(y_{i}-{\hat{y}}_{i}\right)}^{2}\;\;}{n}}\;\;$$

#### 3.6.3. Mean Absolute Percentage Error (MAPE)

Like the MAE, the mean absolute percentage error (MAPE) [69] measures the accuracy of the predicted values with adjusted values to express it as a percentage unit. On average, it indicates the distance between the corresponding actual values and predicted values. The metric tends to be robust to outliers since it uses the absolute values of the error. A score closer to zero indicates a better model. The MAPE can be calculated using Equation (5).

(5) $$\;\mathrm{MAPE}=\frac{1}{n}\;\sum_{i=1}^{n}\left|\frac{y_{i}-{\hat{y}}_{i}}{y_{i}}\right|\;\;\;\;\;\;$$

#### 3.6.4. Coefficient of Determination (R2 score)

The coefficient of determination (R2) metric [70] signifies the association between the predicted values and the actual values. Like MAPE, the R2 score is commonly used as it is expressed in a percentage range and, hence, is more intuitive in results interpretation. A value close to 1 indicates an excellent performance of the model. Equation (6) shows how to calculate the R2 score of a given dataset:

(6) $$\;\;\;\;\;R2=1-\;\frac{\sum^{\;}{\left(y_{i}-\hat{y}\right)}^{2}\;}{\sum^{\;}{\left(y_{i}-\bar{y}\right)}^{2}\;}\;\;\;\;\;\;\;$$

where $\hat{y}$ is the predicted value of y and $\bar{y}$ is the mean value of y.

## 4. Results and Discussion

In this section, a detailed analysis of the result is presented. Our experiments were conducted on a computer machine with the following specification: an Intel Core-i7-7 CPU, 16 GB RAM, and 8 GB NVIDIA GeForce GTX 1060. We ran our experiments on a Windows 10 operating system.

### 4.1. Hyperparameter Optimization

During hyperparameter optimization, a set of hyperparameters for each model was declared to build the search space, as shown in Table 3. We then used the MSE as an objective function, where, for each of the determined configurations, an MSE of the validation set was used to rank the model performance. A set of 100 trials was run for every single model, and finally, the model with the smallest MSE was selected as the best model to generate the results on the test set.

### 4.2. Prediction of Waste Generation Using ARIMA Model

The autoregressive integrated moving average (ARIMA) has been used as one of the classical statistical approaches to forecast time series data [71]. The model tries to draw relationships between the target values and their lagged observations. The algorithm attempts to convert time series data to stationary data by subtracting observation of the previous time step and using the residual and observation between lagged observations. In this study, the ARIMA model was applied to establish a baseline performance for the proposed method. An open-source library (i.e., pmdarima) was used to find the optimal values for the ARIMA model (https://alkaline-ml.com/pmdarima/index.html, accessed on 4 March 2022).

Despite optimizing different model parameters, the performance of the ARIMA model was relatively low compared to all machine learning models. For example, the model achieved an R2 score of around −0.708 and a MAPE of 0.99, suggesting that the model is arbitrarily worse. A similar pattern was observed when looking at other metrics (Table 4).

### 4.3. Prediction of Waste Generation Using NARX Model

The non-linear autoregressive exogenous model (NARX) is a class of neural networks used to model non-linear time-series data [36]. The network consists of recurrent feedback from other network layers and is used in the input layer. An in-depth explanation of the network can be found in [72]. Like the ARIMA model, NARX was also applied to establish a baseline performance for the proposed model. A three-layered ANN was used as an estimator, with the model achieving an R2 score of 0.61. Compared to the ARIMA model, NARX performed better; however, the performance was considerably lower compared to other machine learning models. Nevertheless, when looking at the MSE, the NARX model had a matching performance with the XGBoosting while performing slightly lower than the SVR model (Table 4).

### 4.4. Prediction of Daily Waste Generation Using Machine Learning Algorithms

Each model was optimized, and their performances were analyzed in contrast to the other models. This approach was used to establish a strong baseline performance against our proposed method. All machine learning models were then utilized to establish the proposed ensemble learner.

#### 4.4.1. Support Vector Regressor

**Figure 2:**
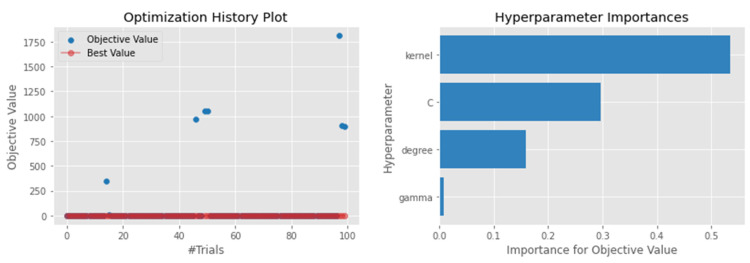
Hyperparameter optimization for the SVR model.

SVR has been widely used in the prediction of waste generation from different studies [53]. After performing model optimization, the R2 score of the model reached 0.69, which suggests that 69% of the variability in the dependent variable is predictable from the independent variable. This performance was achieved when using a ‘c’ value of 2.98 with a radial basis function (RBF). However, the model’s performance was relatively low compared to other algorithms. For example, the MAE was only 0.093, which is on par with other models, such as ANN and XGBoosting. As shown in Figure 2, the kernel and the ‘c’ value were the most important hyperparameters to tune when building a waste prediction model. Figure 2 confirms the importance of using Optuna optimization, as the algorithm only focused on a search space with the possibility to improve the model performance.

#### 4.4.2. XGBoosting

**Figure 3:**
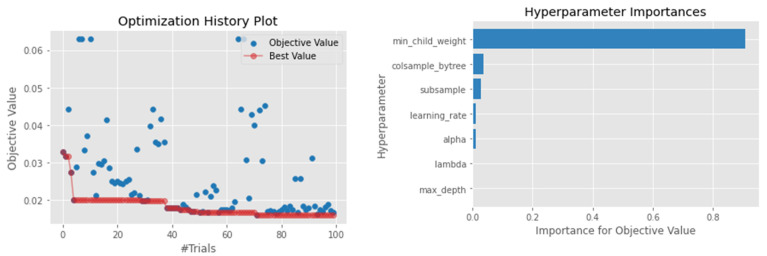
Hyperparameter optimization for the XGBoosting model.

The performance of the XGBoosting was low compared to most of the models. For example, the R2 score was only 0.67, which is the lowest except for the classical models. A similar pattern was observed when looking at both the MSE and the RMSE, which were 0.015 and 0.122, respectively. This may be attributed to a large model parameter that needs to be tuned. The best performance of the model was achieved by using a learning rate of 0.048, a minimum child node weight of 10.289, and a maximum tree depth of 10. As shown in Figure 3, the minimum child node weight was the most important parameter to predict waste generation in this study. Although there was a large search space for the model, Optuna could traverse most potential good candidate configurations, as shown in Figure 3.

#### 4.4.3. LightGBM

**Figure 4:**
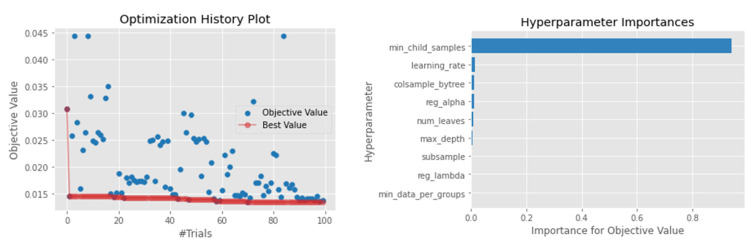
Hyperparameter optimization for the XGBoosting model.

Among the investigated models, LightGBM was among the top-performing algorithms. The model achieved a MAE of 0.08 and a MSE of 0.011, ranking as the second-best model apart from the ensemble models (Table 4). The R2 score of 0.746 was also far better than other algorithms, such as the SVR and XGBoosting, which gave an R2 score of 0.692 and 0.67, respectively. Despite having a fast-training time compared to XGBoosting, the results suggest that LightGBM can be used as a good baseline model when forecasting waste generation. When optimizing Optuna, the most crucial hyperparameter was the minimum child samples used for splitting the node, which was 14 samples (Figure 4). Figure 4 also confirms that, despite having many hyperparameters to tune, the Optuna algorithm was able to find the best hyperparameter configuration even on a limited run.

#### 4.4.4. Random Forest (RF)

**Figure 5:**
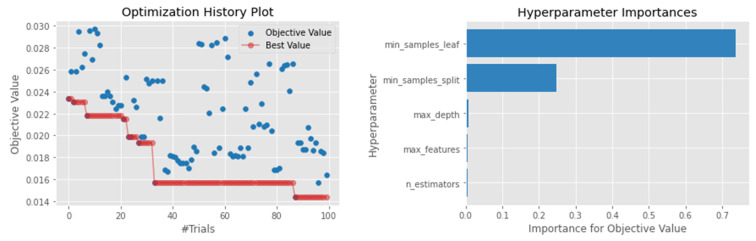
Hyperparameter optimization for the RF model.

Despite being an ensemble technique, the performance of the RF model was also low compared to the top-performing models, such as the LightGBM and KNN. The model achieved an MSE of 0.0128 and a MAPE of 0.382, which is better than the SVR and XGBoosting. A similar result was obtained when looking at the MAE and R2 score (0.085 and 0.7143, respectively), suggesting that around 71% of the dependent variable variability is predictable from the independent variable. Similar to the LightGBM, a minimum sample of a leaf node and minimum samples required to split a node were the most important hyperparameters, obtaining a value of 2 and 7, respectively. Figure 5 shows the hyperparameter importance and the possible configuration explored by the Optuna algorithm. Surprisingly, the number of estimators in most of the tree-based models had a minimum impact.

#### 4.4.5. Artificial Neural Network

**Figure 6:**
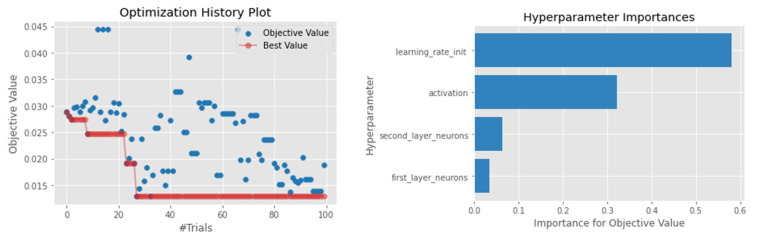
Hyperparameter optimization for the ANN model.

After the initial experimentation, our study focused on optimizing a three-layer neural network. Even though the ANN performed better than the SVR and XGBoosting algorithms in terms of the MAE (Table 4), the performance of SVR was slightly better when using the MSE and R2 scores. Hence, the ANN tends to produce a larger error compared to the SVR. The learning rate and the type of activation used turned out to be the most important hyperparameters to tune in the case of ANN (Figure 6).

#### 4.4.6. K-Nearest Neighbors

**Figure 7:**
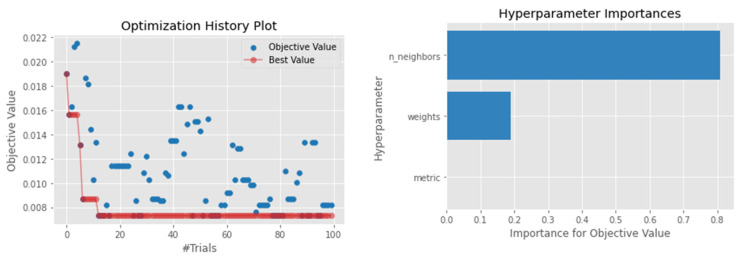
Hyperparameter optimization for the KNN model.

Even though the KNN model was rarely used in the reviewed literature [73], our results suggest that the KNN is among the top-performing models when considering different metrics. For example, the model achieved an MAE of 0.0652, which was the second-ranked individual algorithm. On the other hand, a similar observation was noted when looking at the MAPE, which was better than the voting ensemble, although lower than the proposed model. Notably, the best performance was achieved when looking at a single nearest neighbor by considering a weighted distance among the neighbors. Furthermore, the number of neighbors and the weighted distance were the most influencing hyperparameters in training the KNN model, as shown in Figure 7.

#### 4.4.7. ExtraTrees (ETS)

**Figure 8:**
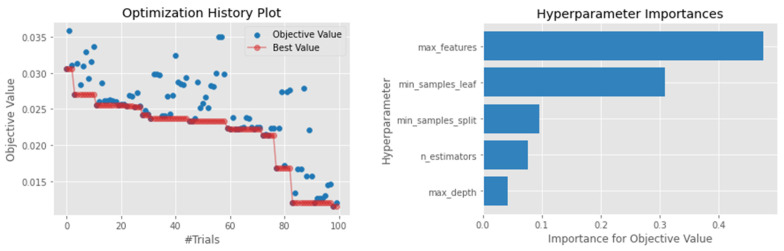
Hyperparameter optimization for the ETS model.

ETS differs from the RF model by randomly selecting features to perform the decision tree’s node split. This feature makes the ETS faster to train and, in some cases, achieve better results compared to the RF [74]. A similar pattern was observed in our study, where the ETS model outperformed the RF across several metrics. For example, looking at the MSE, ETS achieved a MSE of 0.0118 compared to the 0.0128 of RF. On the other hand, the R2 score value was also higher than that of SVR, RF, ANN, and XGBoosting, while matching the KNN and LightGBM, with a value of 0.7368. Similar to most tree-based ensembles, the number of features used to split the node and the minimum number of samples on the leaf node proved to be vital in optimizing the ETS model performance (Figure 8).

### 4.5. Prediction of Daily Waste Generation Using the Proposed Ensemble Model

To obtain the final score of the proposed ensemble model, a combined output of the individual optimized models was performed. These models included a SVR, XGBoosting, ANN, RF, ETS, LightGBM, and KNN model. We first generated an ensemble result by averaging the results of the individual optimized models and measuring their performance against the test set. The performance based on the R2 score, MSE, and RMSE suggests a significant performance improvement over the individual models. For example, when comparing with the KNN model, the ensemble’s performance improved by 0.04 over the KNN model. However, the KNN model was still better when looking at both the MAE and MAPE, with a value of 0.0652 and 0.2816 compared to 0.073 and 0.331. This result suggests that ensemble learning based on averaged results may not reflect a true performance improvement when analyzing different metrics.

In the next step, we used a simple linear regression model as a meta learner to train the model using the predicted output of individual optimized models. As seen from the results in Table 4, there is a consistent improvement in the model performance across all performance metrics. Furthermore, when comparing the performance of the proposed model with individual models, significant improvements were observed. For instance, the R2 score was 0.802 in the ensemble model, in comparison to the 0.748 observed in the KNN model (Table 4). The overall results suggest that using the proposed meta regressor to ensemble results from individual models can have a significant performance advantage over the simple ensemble of averaging the results.

**Table 4:** Performance comparison between the proposed ensemble method and existing ML approaches.

Model	MAE	MSE	RMSE	MAPE	R2 Score
SARIMA [15]	0.191	0.061	0.247	0.99	−0.708
NARX neural network [75]	0.0850	0.014	0.119	0.834	0.612
LightGBM [73]	0.0796	0.0114	0.1066	0.3557	0.7462
KNN [76]	0.0652	0.0112	0.1060	0.2816	0.7489
SVR [77]	0.0927	0.0138	0.1174	0.4015	0.6918
ETS [33]	0.0817	0.0118	0.1085	0.3490	0.7368
RF [78]	0.0850	0.0128	0.1131	0.3822	0.7143
XGBoosting [79]	0.0898	0.0148	0.1215	0.4044	0.6700
ANN [80]	0.0895	0.0141	0.1186	0.3784	0.6854
Ensemble (Average ensemble)	0.073	0.010	0.098	0.331	0.787
Proposed Ensemble (Meta model)	**0.059**	**0.009**	**0.094**	**0.263**	**0.802**

From the above results, we have observed that machine learning algorithms can develop an effective predictive model to forecast future waste generation. We analyzed several models commonly used in the literature and discovered that there is no single best model that performed well across the dataset. For example, despite the ANN model being the most frequently used, our study showed that a simple algorithm such as KNN can perform better than more complex models and should always be included in the evaluation process. On the other hand, despite ensemble learning performing well compared to classical algorithms, they were still outperformed by the KNN algorithm. Nevertheless, the proposed ensemble strategy proved to be more beneficial and outperformed other ensemble models by a considerable margin.

## 5. Theoretical and Managerial Implications

Waste generation forecasting is a significant research challenge across academic societies, and investigating this challenge helps to create a clear insight into the underlying factors impacting the prediction of waste generation in real-world scenarios. In the past, various techniques have been developed to solve this issue, but none of them managed to establish a breakthrough solution. Therefore, there is a new direction of unitizing machine learning solutions to efficiently predict and manage waste. Our research adds to the literature on classification approaches for the prediction of solid waste by providing an ensemble model that outperforms the existing state-of-the-art approaches of household solid waste generation.

While there are various theoretical implications of our solution for the prediction of household solid waste, the method can be used by other researchers in the field for testing and extension. Furthermore, the ensemble model shows promising performance scores, so it can be compared and validated against other ML models in a broader context than just the household scenario exemplified in this study. Even though we evaluated the model critically, further testing across a variety of metrics and scenarios (e.g., datasets, timeframes, cities) is advisable. Researchers may enhance the model with respect to its performance accuracy and prediction of other types of waste. Finally, the model is based on a data-driven approach, so it can be easily combined with other similar models to produce more accurate waste generation predictions in the future.

The managerial implications of our method are substantial. By predicting the amount of solid waste per household with a high precision, urban city decision-makers can create faster and cost-effective routing plans for their collection fleet. These plans would be more efficient in terms of time, cost, and human capital than the traditional approaches. Cities across the globe spend hundreds of millions in their currency for the annual costs of waste collection and disposal. Deploying our method may reduce the number of trips of trucks, thereby minimizing the total number of trucks, drivers, and gasoline. Consequently, we expect a reduction in the traffic congestion and air pollution caused by the waste collection trucks.

The ensemble model has the capability of learning over time, so, inevitably, we will reach the point of minimizing the number of sensors (e.g., in the case of smart bins) to a cost-effective level. It is a data-driven approach, so predictions over one part of a city could be scaled to another part or neighborhood with similar characteristics. Moreover, the waste management authorities can integrate our solution with other initiatives related to recycling and zero solid waste. Last but not least, the method efficiency will boost the trust of local authorities in financing the projects related to the classification of waste for better prediction approaches in the future.

## 6. Conclusions and Future Works

The prediction of household waste generation is an overarching task for waste management authorities across urban cities, as it assists in a better planning and management of waste generation. Due to the limitation of statistical models, machine learning techniques have shown a better predictive power when sufficient data are available. However, finding the right model is not a trivial task, as the models’ behavior differs across a range of data, presenting a challenge in model selection. This study developed an ensemble method based on a meta regressor to predict the weekly household waste generation in urban cities. The ensemble method consists of an optimized mixture of machine learning models with different learning strategies. More precisely, we applied an optimization to the hyperparameter configurations of the single machine learning models using the Optuna algorithm, which is known to apply an efficient sampling and pruning strategy to the search space. Next, we trained our novel meta linear regressor using the outputs of the optimized single learners. Although the solid waste dataset that was used for training and testing was limited with respect to the predictive features, the performance results of our ensemble learning model exceeded that of the single optimized models and average ensemble results.

Our results suggest that the proposed ensemble model outperforms existing models for predicting household waste generation when evaluated across various metrics. Moreover, individual models, such as K.N.N, LightGBM, and ETS, can also give a good baseline performance, as they outperform popular machine learning models, such as the SVR, RF, and ANN. Our study also suggests that it is crucial to evaluate models across different metrics, as focusing on individual metrics may not reflect the correct performance improvements. This is the first study to propose a meta regressor as an ensemble technique for predicting household waste generation from the existing literature. The limitations of our work include the use of a single dataset to validate the proposed model and prediction of weekly solid waste.

As part of the future work, we plan to examine the effects of the homogeneous and heterogeneous ensemble when used with different lag estimations. This will further shed light on the performance improvements that may induce the adoption and use of ensemble models over a single model for predicting solid waste generation. Furthermore, we intend to extend our testing of the ensemble model with various types of time-series datasets (e.g., monthly, daily, hourly) collected from different cities of diverse characteristics.

## Figures and Tables

**Table 1:** Summary of main research weaknesses/gaps in smart waste management.

Article	Strengths	Weaknesses
A multi-layer LoRaWAN infrastructure [38]	A LoRaWAN-based architecture with three layers of implementation is proposed. Fill level measurements, vandalism detection, video surveillance, user privacy, and security. Machine-learning-based image detection mechanism.	The study does not predict fill levels based on waste generation patterns. The route prediction and optimization mechanisms are not discussed. The study does not focus on household waste generation or prediction.
Use of IoT to enable solid waste management [39]	An architecture is presented to monitor the fill levels of smart bins. A GUI-enabled interface is provided for authorities to monitor the fill level. A prototype is implemented and tested.	No fill-level predictions. Route selection and prediction are not presented. Waste generation patterns are not explored.
Prediction of plastic waste generation using SHAP [26]	A neural network (ANN) model is proposed to anticipate the generation of plastic waste. Shapley additive explanation (i.e., SHAP) analysis is applied to interpret the outcomes.	The study focuses on plastic waste generation only. The exclusive target of the study is the European Union. The study does not focus on household waste generation patterns.
Forecasting of regional waste generation [15]	Demographic and socioeconomic variables are used to predict MSW generation and diversion. A real dataset from 2001 to 2014 is utilized. A decision tree and artificial neural network are used to forecast annual waste generation.	The analysis in the study is based on a dataset that is limited to Canada. The specific validity of the analysis is not proven on different datasets or regions with different variables such as demographics, age, income level, etc.
Application of deep neuroevolution for the prediction of waste generation [25]	Waste generation predictions are made based on real-world conditions. Authors utilize a deep neuroevolutionary algorithm that helps to automatically design a recurrent neural network for predicting waste filling levels from waste containers. Authors validate their approach by utilizing real-world scenarios and implementations.	The study focuses on individual metrics that may not reflect the actual performance improvements. Parameters related to waste generation is not discussed, such as demographics, social economics, age, etc.

**Table 2:** A conceptual comparison of single prediction models for smart waste management.

Algorithm		Advantages	Disadvantages
LightGBM	Family of gradient boosting framework that uses a tree-based algorithm	Fast and efficient. Low memory usage. Better performance than most boosting algorithms. Works well with a large dataset.	Prone to overfitting on a smaller dataset.
KNN	Selects k-closest examples from the dataset	Intuitive and straightforward to understand. Constantly changes with the new data.	Does not scale well with a large dataset. Sensitive to outliers.
SVR	Selects the best hyperplane that maximizes separability between classes	It is effective in high dimensional spaces. The algorithm is memory efficient.	Does not scale well when using an extensive dataset.
ETS	An ensemble of decision trees that performs a random split of the node	Extremely fast compared to the RF. Works better than the RF when having a large dataset.	The model may have high variance as it performs random splits.
RF	An ensemble of decision trees that uses a greedy approach to achieve the best split	Reduces the variance of the model and overfitting. Gives information about essential features from the data.	It is computationally expensive compared to other algorithms.
XGBoosting	An ensemble of decision trees that uses a gradient boosting framework	Can handle missing data with its in-built features. Works well on small and medium-sized data.	Very sensitive to outliers. Does not scale well when using massive datasets.
ANN	Inspired by the human brain, ANN consists of several input and output layers that can extract patterns from given data	Ability to learn and model complex relationships. Robust to noisy training data.	Works only on numerical data. Hard to obtain the optimal results.

**Table 3:** Search space configuration for hyperparameter optimization using Optuna.

Algorithm	Search Space
SVR	Kernel = [‘rbf’,’poly’,’linear’,’sigmoid’] C = float (0.1, 3.0) Gamma = [‘auto’,’scale’] Degree = int (1, 3)
XGboost	max_depth = int (4, 12) learning_rate = log uniform (0.005, 0.05) colsample_bytree = log uniform (0.2, 0.6) subsample = log uniform (0.4, 0.8) alpha = log uniform (0.01, 10.0) lambda = log uniform (1 × 10^−8^, 10.0) gamma = log uniform (1 × 10^−8^, 10.0) min_child_weight = log uniform (10, 1000)
LightGBM	reg_alpha = log uniform (1 × 10^−3^, 10.0) reg_lambda = log uniform (1 × 10^−3^, 10.0) colsample_bytree = [0.3, 0.4, 0.5, 0.6, 0.7, 0.8, 0.9, 1.0] subsample = [0.4, 0.5, 0.6, 0.7, 0.8, 1.0] learning_rate = [0.006, 0.008, 0.01, 0.014, 0.017, 0.02] max_depth = [10, 20, 100] num_leaves = int (1, 1000) min_child_samples = int (1, 300) cat_smooth = int (1, 100)
RF	n_estimators =_int (low = 100, high = 1000) max_depth = float (4, 50) min_samples_split = int (2.0, 150.0) min_samples_leaf = int (2.0, 60.0) max_features = [“auto”, “sqrt”, “log2”]
ANN	learning_rate_init = float (0.0001, 0.1, step = 0.005) first_layer_neurons = int (10, 100, step = 10) activation = [‘identity’, ‘tanh’, ‘relu’]
KNN	n_neighbors = int (1, 30) weights = [‘uniform’, ‘distance’] metric = [‘euclidean’, ‘manhattan’, ‘minkowski’]
Extreme Trees	n_estimators = int (low = 100, high = 1000) max_depth = float (4, 50) min_samples_split = int (2.0, 150.0) min_samples_leaf = int (2.0, 60.0) max_features = [“auto”, “sqrt”, “log2”]

## Data Availability

The datasets analyzed or used during the study and source code developed are available at: https://github.com/anamoun/smartwastegeneration (accessed on 29 March 2022).

## References

[1] Worldbank Urban Population. https://data.worldbank.org/indicator/SP.URB.TOTL.IN.ZS.

[2] United Nations. https://www.un.org/development/desa/en/news/population/2018-revision-of-world-urbanization-prospects.html.

[3] Worldbank Trends in Solid Waste Management. https://datatopics.worldbank.org/what-a-waste/trends_in_solid_waste_management.html.

[4] Zanella A., Bui N., Castellani A., Vangelista N., Zorzi M. (2014). Internet of Things for smart cities. IEEE Internet Things J..

[5] Pardini K., Rodrigues J.J., Diallo O., Das A.K., de Albuquerque V.H.C., Kozlov S.A. (2020). A Smart Waste Management Solution Geared towards Citizens. Sensors.

[6] Mdukaza S., Isong B., Dladlu N., Abu-Mahfouz A.M. Analysis of IoT-enabled solutions in smart waste management. Proceedings of the IECON 2018—44th Annual Conference of the IEEE Industrial Electronics Society.

[7] Wu H., Yang B., Tao F. (2020). Optimization of vehicle routing for waste collection and transportation. Int. J. Environ. Res. Public Health.

[8] Pardini K., Rodrigues J.J.P.C., Kozlov S.A., Kumar N., Furtado V. (2019). IoT-Based Solid Waste Management Solutions: A Survey. J. Sens. Actuator Netw..

[9] Ali T., Irfan M., Alwadie A.S., Glowacz A. (2020). IoT-Based Smart Waste Bin Monitoring and Municipal Solid Waste Management System for Smart Cities. Arab. J. Sci. Eng..

[10] Folianto F., Low Y.S., Yeow W.L. Smartbin: Smart waste management system. Proceedings of the 2015 IEEE Tenth International Conference on Intelligent Sensors, Sensor Networks and Information Processing (ISSNIP).

[11] Shyam G.K., Manvi S.S., Bharti P. Smart waste management using Internet-of-Things (IoT). Proceedings of the 2nd International Conference on Computing and Communications Technologies.

[12] Mahajan S.A., Kokane A., Shewale A., Shinde M., Ingale S. (2017). Smart waste management system using IoT. Int. J. Adv. Eng. Res. Sci..

[13] Gupta P.K., Shree V., Hiremath L., Rajendran S. (2019). The Use of Modern Technology in Smart Waste Management and Recycling: Artificial Intelligence and Machine Learning. Advances in Intelligent Information and Database Systems.

[14] Bakhshi T., Ahmed M. Iot-Enabled Smart City Waste Management Using Machine Learning Analytics. Proceedings of the 2018 2nd International Conference on Energy Conservation and Efficiency (ICECE).

[15] Kannangara M., Dua R., Ahmadi L., Bensebaa F. (2017). Modeling and prediction of regional municipal solid waste generation and diversion in Canada using machine learning approaches. Waste Manag..

[16] Medvedev A., Fedchenkov P., Zaslavsky A., Anagnostopoulos T., Khoruzhnikov S. Waste management as an IoT-enabled service in smart cities. Proceedings of the 15th International Conference, NEW2AN 2015, and 8th Conference ruSMART 2015.

[17] Fatimah Y.A., Govindan K., Murniningsih R., Setiawan A. (2020). Industry 4.0 based sustainable circular economy approach for smart waste management system to achieve sustainable development goals: A case study of Indonesia. J. Clean. Prod..

[18] Anagnostopoulos T., Zaslavsky A., Kolomvatsos K., Medvedev A., Amirian P., Morley J., Hadjiefthymiades S. (2017). Challenges and Opportunities of Waste Management in IoT-enabled Smart Cities: A Survey. IEEE Trans. Sustain. Comput..

[19] Fallavi K.N., Kumar V.R., Chaithra B.M. Smart waste management using Internet of Things: A survey. Proceedings of the 2017 International Conference on I-SMAC (IoT in Social, Mobile, Analytics and Cloud) (I-SMAC).

[20] Londres G., Filipe N., Gama J., Cellier P., Driessens K. (2019). Optimizing Waste Collection: A Data Mining Approach. ECML PKDD 2019: Machine Learning and Knowledge Discovery in Databases, Proceedings of the Joint European Conference on Machine Learning and Knowledge Discovery in Databases, Würzburg, Germany, 16–20 September 2019.

[21] Camero A., Toutouh J., Ferrer J., Alba E. (2018). Waste generation prediction in smart cities through deep neuroevolution. Ibero-American Congress on Information Management and Big Data.

[22] Sheng T.J., Islam M.S., Misran N., Baharuddin M.H., Arshad H., Islam M.R., Chowdhury M.E.H., Rmili H., Islam M.T. (2020). An internet of things based smart waste management system using LoRa and tensorflow deep learning model. IEEE Access.

[23] Ahmed N., Atiya A., Gayar N., El-Shishiny H. (2010). An Empirical Comparison of Machine Learning Models for Time Series Forecasting. Econom. Rev..

[24] Ferrer J., Alba E. (2019). BIN-CT: Urban waste collection based on predicting the container fill level. Biosystems.

[25] Camero A., Toutouh J., Ferrer J., Alba E. (2019). Waste generation prediction under uncertainty in smart cities through deep neuroevolution. Rev. Fac. Ing. Univ. Antioq..

[26] Fan Y.V., Jiang P., Tan R.R., Aviso K.B., You F., Zhao X., Lee C.T., Klemeš J.J. (2021). Forecasting plastic waste generation and interventions for environmental hazard mitigation. J. Hazard. Mat..

[27] Jassim M.S., Coskuner G., Zontul M. (2022). Comparative performance analysis of support vector regression and artificial neural network for prediction of municipal solid waste generation. Waste Manag. Res..

[28] Kontokosta C.E., Hong B., Johnson N.E., Starobin D. (2018). Using machine learning and small area estimation to predict building-level municipal solid waste generation in cities. Comput. Environ. Urban Syst..

[29] Yang L., Zhao Y., Niu X., Song Z., Gao Q., Wu J. (2021). Municipal Solid Waste Forecasting in China Based on Machine Learning Models. Front. Energy Res..

[30] Kulisz M., Kujawska J. (2020). Prediction of municipal waste generation in Poland using neural network modeling. Sustainability.

[31] Flores C.A.R., da Cunha A.C., Cunha H.F.A. (2022). Solid waste generation indicators, per capita, in Amazonian countries. Environ. Sci. Pollut. Res..

[32] Elshaboury N., Mohammed Abdelkader E., Al-Sakkaf A., Alfalah G. (2021). Predictive Analysis of Municipal Solid Waste Generation Using an Optimized Neural Network Model. Processes.

[33] Rathod T., Hudnurkar M., Ambekar S. (2020). Use of Machine Learning in Predicting the Generation of Solid Waste. Pjaee.

[34] Meza J.K.S., Yepes D.O., Rodrigo-Ilarri J., Cassiraga E. (2019). Predictive analysis of urban waste generation for the city of Bogotá, Colombia, through the implementation of decision trees-based machine learning, support vector machines and artificial neural networks. Heliyon.

[35] Abbasi M., El Hanandeh A. (2016). Forecasting municipal solid waste generation using artificial intelligence modelling approaches. J. Waste Manag..

[36] Kumar S., Kumar R. (2021). Forecasting of municipal solid waste generation using non-linear autoregressive (NAR) neural models. Waste Manag..

[37] Ali S., Ahmad A. (2019). Forecasting MSW generation using artificial neural network time series model: A study from metropolitan city. SN Appl. Sci..

[38] Baldo D., Mecocci A., Parrino S., Peruzzi G., Pozzebon A. (2021). A Multi-Layer LoRaWAN Infrastructure for Smart Waste Management. Sensors.

[39] Vishnu S., Ramson S., Senith S., Anagnostopoulos T., Abu-Mahfouz A., Fan X., Srinivasan S., Kirubaraj A. (2021). IoT-Enabled Solid Waste Management in Smart Cities. Smart Cities.

[40] Balaji S., Nathani K., Santhakumar R. (2019). IoT Technology, Applications and Challenges: A Contemporary Survey. Wirel. Pers. Commun..

[41] Razmjoo A., Gandomi A., Mahlooji M., Astiaso Garcia D., Mirjalili S., Rezvani A., Ahmadzadeh S., Memon S. (2022). An Investigation of the Policies and Crucial Sectors of Smart Cities Based on IoT Application. Appl. Sci..

[42] Salehi-Amiri A., Akbapour N., Hajiaghaei-Keshteli M., Gajpal Y., Jabbarzadeh A. (2022). Designing an effective two-stage, sustainable, and IoT based waste management system. Renew. Sustain. Energy Rev..

[43] Claire N.U.M., Ngend L. (2022). IOT Based Waste Management for Smart City, Case of Musanze City. Int. J. Progress. Sci. Tech..

[44] Shukla S., Hait S. (2022). Smart waste management practices in smart cities: Current trends and future perspectives. Advanced Organic Waste Management: Sustainable Practices and Approaches.

[45] John J., Varkey M.S., Podder R.S., Sensarma N., Selvi M., Santhosh Kumar S.V.N., Kannan A. (2022). Smart Prediction and Monitoring of Waste Disposal System Using IoT and Cloud for IoT Based Smart Cities. Wirel. Pers. Com..

[46] Tasnim R.S. (2014). Ensemble Classifiers and Their Applications: A Review. Int. J. Comput. Trends Technol..

[47] Chongomweru H., Kasem A. (2021). A novel ensemble method for classification in imbalanced datasets using split balancing technique based on instance hardness (sBal_IH). Neural Comput. Appl..

[48] GitHub Smart Waste Generation. https://github.com/anamoun/smartwastegeneration.

[49] Caiafa C.F., Sun Z., Tanaka T., Marti-Puig P., Solé-Casals J. (2021). Machine Learning Methods with Noisy, Incomplete or Small Datasets. Appl. Sci..

[50] Brownlee J. (2019). Basic Feature Engineering with Time Series Data in Python. Machine Learning Mastery. https://machinelearningmastery.com/basic-feature-engineering-time-series-data-python.

[51] Masini R.P., Medeiros M.C., Mendes E.F. (2021). Machine learning advances for time series forecasting. J. Econ. Sur..

[52] Surakhi O., Zaidan M.A., Fung P.L., Hossein Motlagh N., Serhan S., AlKhanafseh M., Ghoniem R.M., Hussein T. (2021). Time-Lag Selection for Time-Series Forecasting Using Neural Network and Heuristic Algorithm. Electronics.

[53] Xia W., Jiang Y., Chen X., Zhao R. (2021). Application of machine learning algorithms in municipal solid waste management: A mini review. Waste Manag. Res..

[54] Fernandez-Delgado M., Cernadas E., Barro S. (2014). Do we Need Hundreds of Classifiers to Solve Real World Classification Problems?. J. Mach. Learn..

[55] Sami K.N., Amin Z.M.A., Hassan R. (2020). Waste Management Using Machine Learning and Deep Learning Algorithms. Int. J. Perceptive Cogn. Comput..

[56] Cubillos M. (2020). Multi-site household waste generation forecasting using a deep learning approach. Waste Manag..

[57] Kumar A., Samadder S.R., Kumar N., Singh C. (2018). Estimation of the Generation Rate of Different Types of Plastic Wastes and Possible Revenue Recovery from Informal Recycling. Waste Manag..

[58] Abbasi M., Rastgoo M.N., Nakisa B. (2019). Monthly and seasonal modeling of municipal waste generation using radial basis function neural network. Env. Prog. Sust. Energy.

[59] Fernández-Delgado M., Sirsat M.S., Cernadas E., Alawadi S., Barro S., Febrero-Bande M. (2019). An extensive experimental survey of regression methods. Neural Netw..

[60] Yang L., Shami A. (2020). On hyperparameter optimization of machine learning algorithms: Theory and practice. Neurocomputing.

[61] Wu J., Chen X.Y., Zhang H., Xiong L.D., Lei H., Deng S.H. (2019). Hyperparameter optimization for machine learning models based on bayesian optimization. J. Electron. Sci. Technol..

[62] Akiba T., Sano S., Yanase T., Ohta T., Koyama M. Optuna: A next-generation hyperparameter optimization framework. Proceedings of the 25th ACM SIGKDD International Conference on Knowledge Discovery & Data Mining.

[63] Schütt M. (2021). Systematic Variation in Waste Site Effects on Residential Property Values: A Meta-Regression Analysis and Benefit Transfer. Env. Res. Econ..

[64] Zheng D., Shen J., Li R., Jian B., Zeng J., Mao Y., Zhaang X., Halder P., Qu M. (2022). Understanding the key factors determining rural domestic waste treatment behavior in China: A meta-analysis. Envi. Sci. Pollut. Res..

[65] Funch O.I., Marhaug R., Kohtala S., Steinert M. (2021). Detecting glass and metal in consumer trash bags during waste collection using convolutional neural networks. Waste Manag..

[66] Zhao L., Pan Y., Wang S., Zhang L., Islam M.S. (2021). Skip-YOLO: Domestic Garbage Detection Using Deep Learning Method in Complex Multi-scenes. Res. Sq..

[67] Karunasingha D.S.K. (2022). Root mean square error or mean absolute error? Use their ratio as well. Inf. Sci..

[68] Kim T., Oh J., Kim N., Cho S., Yun S.Y. (2021). Comparing Kullback-Leibler Divergence and Mean Squared Error Loss in Knowledge Distillation. arXiv.

[69] Morresi N., Casaccia S., Sorcinelli M., Arnesano M., Uriarte A., Torrens-Galdiz J.I., Revel G.M. (2021). Sensing Physiological and Environmental Quantities to Measure Human Thermal Comfort Through Machine Learning Techniques. IEEE Sens. J..

[70] Chicco D., Warrens M.J., Jurman G. (2021). The coefficient of determination R-squared is more informative than SMAPE, MAE, MAPE, MSE and RMSE in regression analysis evaluation. PeerJ Comput. Sci..

[71] Marandi F., Ghomi S.M.T.F. Time series forecasting and analysis of municipal solid waste generation in Tehran city. Proceedings of the 2016 12th International Conference on Industrial Engineering (ICIE).

[72] Carbonera L.F.B., Pinheiro B.D., Karnikowski D.D.C., Alberto F.F. (2021). The non-linear autoregressive network with exogenous inputs (NARX) neural network to damp power system oscillations. Int. Trans. Electr. Energy Syst..

[73] Guo H.-N., Wu S.-B., Tian Y.-J., Zhang J., Liu H.-T. (2021). Application of machine learning methods for the prediction of organic solid waste treatment and recycling processes: A review. Bioresour. Technol..

[74] Geurts P., Ernst D., Wehenkel L. (2006). Extremely Randomized Trees. Mach. Learn..

[75] Boussaada Z., Curea O., Remaci A., Camblong H., Mrabet Bellaaj N. (2018). A nonlinear autoregressive exogenous (narx) neural network model for the prediction of the daily direct solar radiation. Energies.

[76] Dubey S., Singh P., Yadav P., Singh K.K. (2020). Household Waste Management System Using IoT and Machine Learning. Procedia Comput. Sci..

[77] Oguz-Ekim P. (2021). Machine Learning Approaches for Municipal Solid Waste Generation Forecasting, Environ. Eng. Sci..

[78] Ghanbari F., Kamalan H., Sarraf A. (2021). An evolutionary machine learning approach for municipal solid waste generation estimation utilizing socioeconomic components. Arab. J. Geosci..

[79] Jayaraman V., Parthasarathy S., Lakshminarayanan A.R., Singh H.K. Predicting the Quantity of Municipal Solid Waste using XGBoost Model. Proceedings of the 2021 Third International Conference on Inventive Research in Computing Applications (ICIRCA).

[80] Vu H.L., Ng K.T.W., Bolingbroke D. (2018). Time-lagged effects of weekly climatic and socioeconomic factors on ANN municipal yard waste prediction models. Waste Manag..

